# Hematological and Biochemical Profile of Patients Infected with *Schistosoma mansoni* in Comparison with Apparently Healthy Individuals at Sanja Town, Northwest Ethiopia: A Cross-Sectional Study

**DOI:** 10.1155/2020/4083252

**Published:** 2020-05-06

**Authors:** Nega Dessie, Wossenseged Lema, Mulugeta Aemero

**Affiliations:** Department of Medical Parasitology, CMHS, University of Gondar, Gondar, Ethiopia

## Abstract

**Background:**

Schistosomiasis is a parasitic disease that resides in the vascular system of vertebrates, causing a chronic, debilitating disease that affects more than 200 million people and 800,000 deaths per year in over 70 countries. This parasite causes liver dysfunction and disorders normal hematological and biochemical profiles in addition to portal vein hypertension syndrome, ascites, and liver fibrosis. The general objective of the current study is to assess hematological and biochemical profiles of patients infected with *Schistosoma mansoni* in comparison with apparently healthy individuals (control group) in Sanja town, northwest Ethiopia.

**Method:**

A comparative cross-sectional study was conducted from February to April 2019 among microscopically confirmed *S. mansoni*-infected patients attending Sanja hospital and apparently healthy (control group) from Sanja town community. A total of 220 participants, 110 from the *S. mansoni*-infected and 110 from the control group, were enrolled using convenient sampling technique. Three grams of stool and six milliliters of blood samples were collected from each study participant. Stool samples were processed using the Kato–Katz technique to determine infection and count parasite density. The blood sample was processed for the analysis of hematological and biochemical profiles using Cell Dyn 1800 (Abbot Hematology, IL, USA) and iChem535 chemistry analyzer, respectively. All data were analyzed using SPSS version 20, and *P* value less than 0.05 was taken as statistically significant.

**Results:**

This study showed that the mean values of serum alanine aminotransferase, aspartate aminotransferase, total protein, total cholesterol, hemoglobin, mean corpuscular hemoglobin concentration, and total white blood cell count were different in the *Schistosoma mansoni*-positive group as compared with the control group with statistically significant value (*P* ≤ 0.05). However, the mean values of blood glucose, red blood cell, packed cell volume, and granulocyte count difference were not statistically significant (*P* ≥ 0.05). The mean value of hemoglobin, red blood cells, blood glucose, mean corpuscular hemoglobin concentration, total protein, total cholesterol, and total white blood cell was significantly dropped in the moderate and heavy *S. mansoni* parasitic load patients as compared with the control group and light *S. mansoni* parasite density patients. However, the mean of AST and ALT progressively elevated as the burden of *S. mansoni* increased*. Conclusion*. Most hematological and biochemical profiles were significantly lower in the *Schistosoma mansoni*-positive group as compared with the control group. Most hematological and biochemical profiles decline significantly as the parasite density increased. Hence, with *Schistosoma* treatment, supportive treatment against hematological and biochemical disorders is recommended.

## 1. Background

Schistosomiasis is a chronic parasitic disease caused by a trematode blood fluke of the genus *Schistosoma* which belongs to the Schistosomatidae family, namely, *Schistosoma mansoni*, *Schistosoma hematobium*, *Schistosoma intercalatum*, *Schistosoma mekongi*, and *Schistosoma japonicum* [1]. Schistosomiasis is the second most widespread human parasitic disease next to malaria [2]. This parasite mostly affects developing countries. It affects more than 200 million people [1] and causes 800,000 deaths per year in over 70 countries [3]. Clinical presentation and pathology of schistosomiasis are associated with the species causing the disease, where it resides, and the intensity of infection [3].

*Schistosoma* pathogenesis is mainly associated with the host's responses to *Schistosoma* egg antigens to form granulomas usually in the intestines and the liver where the eggs are trapped [4]. Schistosome eggs that do not successfully pass through the intestinal mucosa towards the lumen are usually carried by the portal vein blood flow to the liver inside small presinusoidal and blocking the vessels elicit strong granuloma [5].

Chronic egg-induced granulomas can lead to chronic hepatitis, portal hypertension, bleeding esophageal varices, liver failure, and eventually may lead to death [6]. Another health impact of *S. mansoni*-infected patients is anemia [7]. The intensity of the infection is a crucial factor which can determine clinical manifestations and severity of the disease, but also, other factors, such as superimposed infections, malnutrition, and genetic background, may play a role [8]. Consequently, schistosomiasis causes diarrhea, fatigue, and anemia at the early stage of infection and portal vein hypertension syndrome, ascites, and liver fibrosis at the later stages [1]. Schistosomiasis also affects the host hematological profiles either by direct ingestion through the gut or aggravation of blood loss through feces by rupturing the blood vessels by the help of the egg spine [9]. A wide range of liver damage has been described in human infections. These include impaired protein synthesis, liver fibrosis [10], and reduced total protein concentrations [11]. Previous study had demonstrated that an adult male and female worm ingested about 100 and 900 nl of blood into their gut per day, respectively [12]. A study conducted in Sudan indicated that most hematological profiles like hemoglobin (Hgb), red blood cell (RBC) count, packed cell volume (PCV), mean corpuscular volume (MCV), mean corpuscular hemoglobin (MCH), mean corpuscular hemoglobin concentration (MCHC), differential leukocyte, and total white blood cell (WBC) count were significantly reduced in *S. mansoni*-infected patients as compared with the control group [13].

Although different epidemiological research studies on *Schistosoma mansoni* are conducted in the current study area, there is no report on hematological and biochemical profiles of patients infected with *S. mansoni*. Hence, the objective of the current study was to assess hematological and biochemical profiles of patients infected with *Schistosoma mansoni* in comparison with apparently healthy individuals (control group) in Sanja town, northwest Ethiopia.

## 2. Methods

### 2.1. Study Area

The study was conducted in Sanja town, located about 792 km away from Addis Ababa in Amhara region, northwest Ethiopia. The area has an altitude of 1,800 m above sea level with N 12°59′E 37°18′ coordinates, and its annual rainfall and average temperature range from 800 to 1,800 mm and 25°C to 42°C, respectively [14]. A total of 162, 250 populations live in this district. There are 9 health centers and one district hospital under this district. More than 162,250 populations have access for health service delivery by Sanja hospital [15].

### 2.2. Study Design and Period

A comparative cross-sectional study was conducted from February 2019 to March 2019 among microscopically confirmed *S. mansoni* patients attending Sanja hospital and apparently healthy individuals (control group) from Sanja town community.

### 2.3. Study Population

All *Schistosoma mansoni*-positive subjects whose age is 5 years and above attending Sanja hospital and apparently healthy subjects in the community were considered as the study population.

### 2.4. Sample Size Determination and Sampling Technique

Double population means (sample size for each group) formula was used. A minimum of 110 participants positive for *S. mansoni* infection and 110 *S. mansoni*-negative control groups were enrolled.

The formula *N* = (*S*_1_^2^ + *S*_2_^2^) (*α*, *β*)/(*U*_1_ − *U*_2_)^2^, was used to calculate sample size where *β* = 0.1 (power 90) and *α* = 5% (confidence interval 95%) two side *t* test *f* (*α*, *β*) = (*Z*_*α*/2_ + *Z*_*β*_)^2^ = (1.96 + 1.28)^2^ = 10.5 for two sided test= (0.22)^2^ + (0.23)^2^ (1.96 + 1.28)^2^/(2.2–2.3)^2^ = 100 for each group 100 study participants needed, total = 200, then 10% nonresponse rate was added 200 *∗* 10/100 = 20. The total sample size was 220, and *U*_1_ and *S*_1_^2^ are the mean and the variance of the control group, respectively. *U*_2_ and *S*_2_^2^ are the mean and the variance of the *S. mansoni*-infected group, respectively [16].

### 2.5. Stool Sample Collection and Processing

#### 2.5.1. Microscopic Diagnosis of *Schistosoma mansoni*

Three grams of the fresh stool sample were collected using clean, dry, and screw-capped plastic containers labeled with unique identification. Stool samples were processed through the direct wet mount for detection of parasites and Kato–Katz technique using a standardized template measuring 41.7 mg of the stool to prepare a thick smear for detection and quantification of the density of *S. mansoni* ova for infected participants [17].

#### 2.5.2. Blood Sample Collection

Six milliliters of venous blood was collected by experienced blood collectors using a sterile disposable plastic syringe after cleaning the venous puncture site with 70% ethanol. Three milliliters of blood was transferred into the ethylene diamine tetra acetic acid (EDTA) test tube for hematological profile analysis. The remaining 3 ml was placed in a plane test tube for analysis of biochemical profiles [18, 19].

### 2.6. Determination of Hematological Parameters

The 3 ml blood in the EDTA tube was dispensed and analyzed by Cell Dyn 1800 (Abbot Hematology, IL, USA) for the determination of complete blood cell count [20].

### 2.7. Serum Biochemical Analysis

Three milliliters of blood in the anticoagulant-free (plane) test tube was allowed to clot at bench top at least for 30 minutes. Then, it was centrifuged at 2,500 revolutions per minute for 4 minutes, and serum was aliquot by an Eppendorf tube. Then, serum was analyzed by using iChem535 chemistry analyzer for serum level of AST, ALT, blood glucose, total protein, and serum total cholesterol [21].

### 2.8. Data Analysis

Data were registered into Statistical Package for Social Sciences (SPSS) version 20 for statistical analysis of mean and standard deviations, while the differences between the study and the control groups were assessed using the independent *t*-test and one-way ANOVA. The *P* value with 95% of confidence interval was used to determine the association between the dependent and independent variables. Statistical significance was considered at a 95% level of confidence and *P* value less than 0.05.

### 2.9. Ethical Considerations

The study was conducted after ethical approval was obtained from the Research and Ethics committee of the School of Biomedical and Laboratory Sciences, College of Medicine & Health Sciences, University of Gondar. Moreover, letter of support was secured from the Sanja district health office and community leader. Before starting the actual data collection, permission was obtained from the hospital director. Additionally, after explaining the purpose, benefits, and the possible risks of the study, informed written consent was obtained from the study participants or their guardians. All laboratory results were kept confidential. Apparently, those positive for parasite and with hematological and biochemical abnormality were linked to the hospital and health center for appropriate treatment. The treatment cost was covered by communicating with the zonal and district health administrators.

## 3. Results

A total of 220 subjects were recruited for this study. Of these, 110 were *S. mansoni*-positive, and the remaining were apparently healthy control group. Age ranged from 8 to 60 years with a mean age of 30.90 ± 9.11 for *S. mansoni* positive and 31.11 ± 13.20 for the control group. Half (110/220) of the participants were males and rural in residence. The majority (69.1%) of *S. mansoni*-positive individuals had a light parasite density (Table 1).

In the current study, the mean total WBC count in the *S. mansoni*-infected subject was low as compared with the apparently healthy control group with a mean and standard deviation of 4.60 ± 1.61 and 7.81 ± 3.44, respectively. Similarly, the mean RBC count was slightly lower in the *S. mansoni*-positive group as compared with the control group, 3.92 ± 0.75 and 4.04 ± .65, respectively (Table 2). The mean Hgb concentration of the *S. mansoni*-positive group and the control group was 11.36 ± .1.89 and 12.58 ± 1.67, respectively (Table 1).

The mean blood glucose level was lower in the *S. mansoni*-infected group than the control group (99.71 ± 18.67 &104.13 ± 16.63), respectively, without a statistically significant association (*P* > 0.05). However, the mean serum total protein was significantly lower in the *S. mansoni*-infected group (68.70 ± 3.20) than the control group (69.76 ± 3.03). The mean serum AST level was elevated in *S. mansoni*-infected individuals (24.84 ± 2.19) compared with the control group (23.86 ± 2.22). The mean ALT level in *S. mansoni*-positive subjects was elevated (22.67 ± 4.66) compared with the control group (21.15 ± 2.27) (Table 2).

In the current study, mean of the most hematological profiles significantly (*P* < 0.05) declines with increasing of parasite intensity. The mean value of HGB, HCT, MCH, MCHC, blood glucose, total protein, total cholesterol, and total WBC was significantly (*P* < 0.05) dropped in the moderately and heavily *S. mansoni*-infected patients compared with the control group and those with light *S. mansoni* egg density (*P* < 0.05). However, the mean value of AST was progressively elevated as the intensity of *S. mansoni* infection increased (*P* < 0.001) (Table 3).

## 4. Discussion

Schistosomiasis remains one of the most important but neglected tropical diseases in Ethiopia [22]. In the current study, the intensity of *S. mansoni* infection was 69.1% light, 28.2% moderate, and 2.7% heavy. This result is nearly similar to the result that is previously reported from Azezo, northwest Ethiopia, 67.8%, 19.8%, and, 3.1% light, moderate, and heavy intensity, respectively [23]. The current study result is quite different from another report from Sanja, northwest Ethiopia, 0% light, 86.2% moderate, and 13.8% heavy intensity of infection [24]. Another study in Gorgora town, northwest Ethiopia, had reported 13.1% light, 7.1% moderate, and 0.3% heavy intensity of infection [25]. There was another incomparable report from southwest Ethiopia, where 57% light, 26.7% moderate, and 16.3% heavy intensity of infection had been reported [26]. The possible difference across the studies might be due to the age of the study participant, mass drug administration programs, immunity of the host, frequency of individual contact with contaminated water bodies, the burden of the adult worms hosted, sample size, and sampling techniques.

In the present study, the *S. mansoni*-positive subjects had significantly (*P* < 0.001) lower mean of total WBC count as compared with the control group. This result is nearly comparable to the results of previously reported findings from a study in Nigeria, 2.5 × 10^3^ ± 0.12 and 8.0 × 10^3^ ± 2.10, *S. mansoni*-positive and the control group, respectively [16]. In the current study, increased intensity of infection also significantly (*P* < 0.05) dwindled the mean total WBC in severely and moderately infected subjects as compared with the mildly infected group. The current study result was quite lower from a report from Sudan whose mean value of WBC in mild (7.5319 × 10^3^) and severe (6.884 × 10^3^) infected patients was high [27]. *Schistosoma mansoni* like other helminth parasites manipulates the host immune response toward their comfortable condition. Therefore, observed changes in circulating total WBC numbers following *S. mansoni* infection are linked to the basic immune response to the parasite and also to induced biased proliferation or decrease in specific immune cells according to the disease stage and density [28]. The current study showed significant (*P* < 0.05) degree elevation of the granulocyte (neutrophil and eosinophil) in the *S. mansoni*-infected subject as compared with the control group. During the acute infections with tissue-migrating larvae or following the sudden release of antigens from parasites dying either spontaneously or following chemotherapy is characterized by *Schistosoma*-induced eosinophil elevation [29].

In the present study, there was no significant (*P* > 0.05) decrease in the mean erythrocytic parameters (the mean values of RBC count and PCV) below the normal range in the *S. mansoni*-infected subject as compared with the control group. The risk is also negatively associated with the burden of the parasite density. This result was slightly lower from a previous study in China with means of RBC 4.64 × 10^6^/UL (*Schistosoma*-positive) and 4.7 × 10^6^/UL (control group) [30]. The decrease in RBC count might be due to the reduction in erythropoiesis in the bone marrow, massive blood consumption by female and male *S. mansoni* worms [9], and enhanced hemolysis of peripheral RBC in the spleen or chronic blood loss through stool [31, 32].

The result from this study showed significantly (*P* < 0.001) dropped mean Hgb concentration with a cutoff point (<12) suggesting for anemia in *S. mansoni*-positive individuals as compared with the control group. This finding is in agreement with a result reported from west Burkina Faso which showed a mean value of HGB (g/dL) 11.57 ± 0.7 6 in *Schistosoma*-positive and 11.86 ± 1.51 in the control group [33]. In the current study, the mean Hgb concentration was also significantly (*P* < 0.001) dropped as the intensity of infection increased. Subjects with heavy infection density were more anemic than those with light and moderate infection. This finding is supported by other findings from western Kenya with a mean Hgb (g/dL) 11.6 in the control group and with light (11.9), moderate (11.3), and (10.9) heavy *Schistosoma mansoni* infection [34]. Another study from Sudan reported mean Hgb (g/dL) value among mild (13.406) and severe (11.692) infections [27].

The decrease in Hgb can be related to the reduction in size of RBC, impaired biosynthesis of heam in the bone marrow, due to reduction in the rate of formation of RBC [35] or can be attributed to chronic blood loss that results from the bleeding induced by migration of worms through the intestinal wall or due to blood consumption by adult schistosomes. In addition, this could be from direct competition with the human host for iron in the intestinal and blood stages of the parasite [36].

In the present study basic results, the mean MCH and MCHC were significantly (*P* < 0.05) lower in the *S. mansoni*-positive group as compared with the control group. This finding was comparable with another recent report from Ghanaian study that showed mean of MCH 23.72 ± 0.43 in the *S. mansoni*-positive group and 26.21 ± 0.31 in the control group and mean of MCHC 31.25 ± 0.27 in the *S. mansoni*-positive group and 32.21 ± 0.19 in the control group [37]. A study in Burkina Faso showed a mean MCH of 26.26 ± 1.83 value in the *S. mansoni*-positive group and 27.12 ± 2.82 in the control group, while the MCHC value was 33.02 ± 0.61 and 32.98 ± 0.72 in the *S. mansoni*-positive and control group, respectively [33].

The results from this present study showed that the mean blood glucose level was not significantly (*P* > 0.05) decreased in the *S. mansoni*-positive group as compared with the control group. Previous study in China reported a mean blood glucose level of 5.33 mmol/L (96 mg/dl) and 5.56 mmol/L (100.1 mg/dl) in the *Schistosoma*-positive group and the control group, respectively [30]. However, those with high intensity of infection have significantly lower glucose level compared with the control study subjects. Structural damage of the host gastrointestinal mucosa can lead to loss and malabsorption of nutrients, reducing food intake, increasing nutrient excretion, vomiting, diarrhea, or altering nutrient metabolism within the body or direct consumption of huge host blood glucose [12].

The current result shows mean of total protein was significantly (*P* < 0.05) declined in the *S. mansoni*-positive group as compared with the control group. This finding agrees with a study from Sudan where a mean total protein of 68 ± 0.1 and 76 ± 0.1 in the *S. mansoni*-positive and control group, respectively, was reported [13]. This result is also supported by another study from Nigeria with a mean value of 50.2 ± 2.10 and 71.0 ± 3.22 in the *S. mansoni*-infected and control group, respectively [16]. The possible reason for reduction of serum protein could be due to impaired protein synthesis [38], a massive amount of plasma protein consumption for the parasite metabolism, development, and growth [39].

The present study showed an elevated mean value of ALT and AST in the *S. mansoni*-positive group as compared with the control group. The previous study from Nigeria reported a mean AST and ALT value in the *S. mansoni*-positive group (25.0 ± 0.63 and 20.1 ± 0.68) and in the control group (6.2 ± 0.18 and 7.1 ± 0.2), respectively [16]. The possible causes might be due to damages on the membrane of the liver, hepatic manifestations originating from the deposition of eggs inside the small vessels of the liver which can lead to an intense inflammatory response and subsequent functional changes, a situation which presumably may be responsible for significant elevation of these circulating liver enzymes [11, 40].

The mean total cholesterol level was significantly (*P* < 0.05) dropped in the *S. mansoni*-infected subject as compared with the control group in the current study. This finding is supported by a study from Brazil, which reported a mean total cholesterol level of 123.90 ± 21.52 and 156.31 ± 41.50 for the *S. mansoni*-positive group and the control group, respectively [41]. The possible cause for reduction of blood total cholesterol in infected hosts might be due to abnormalities in lipid metabolism, and since *Schistosoma* did not synthesize cholesterol *de novo*, it is completely dependent on the host cholesterol and fatty acids [42].

## 5. Conclusion

In the present study, the mean concentrations of ALT, AST, Hgb, MCH, MCHC, total protein, total cholesterol, and WBC count were significantly altered in the *S. mansoni*-infected patients as compared with the control group.

Severely parasitized patients showed a significant reduction in their mean MCH, MCHC, Hgb, blood glucose, total protein, and total cholesterol when compared with mildly and moderately infected study subjects. In general, the study showed that the hematological and biochemical profiles of patients infected with *Schistosoma mansoni* were impaired compared with the normal level and control group. Therefore, schistosomiasis control programs should be strengthened in the study area and elsewhere to decrease its transmission. Other than treatment with Praziquantel, supportive treatment to alleviate health impairment due to hematological and biochemical disorders induced by *S. mansoni* infection should be considered.

## Figures and Tables

**Table 1:** Sociodemographic characteristics and independent *t* comparisons mean difference between hematological profiles in *S. mansoni*-positive and control study participants at Sanja town, northwest Ethiopia, 2019.

Parameter	*S. mansoni* positive (*N* = 110)	Control group (*N* = 110)	*P* value
	Mean ± SD	Mean ± SD	
Mean age and standard division	30.90 ± 9.11	31.11 ± 13.20	
Sex			
Male	55 (50%)	55 (50%)	
Female	55 (50%)	55 (50%)	
Residence			
Urban	55 (50%)	55 (50%)	
Rural	55 (50%)	55 (50%)	
Parasite intensity			
Light	76 (69.1%)	0%	
Moderate	31 (28.2)	0%	
Heavy	3 (2.7%)	0%	
WBC (×10^3^/*μ*L)	4.60 ± 1.61	7.81 ± 3.44	<0.001
L (×10^3^/*μ*L)	1.50 ± 0.61	2.48 ± 1.32	<0.001
Mixed (×10^3^/*μ*L)	0.55 ± 0.53	1.55 ± 1.29	<0.001
Granulocyte (×10^3^/*μ*L)	2.63 ± 1.27	4.03 ± 2.07	<0.001
RBC (×10^6^/*μ*L)	3.92 ± 0.75	4.04 ± 0.65	0.208
Hgb (g/dl)	11.36 ± 0.1.89	12.58 ± 1.67	<0.001
PCV (%)	34.14 ± 5.38	37.51 ± 0.5.11	0. 697
MCV (*μ*m^3^)	92.78 ± 7.78	87.91 ± 5.02	<0.001
MCH (g/dL)	28.90 ± 3.45	30.35 ± 2.95	0. 001
MCHC (Pg)	30.10 ± 4.10	34.80 ± 1.00	<0.001

**Table 2:** Independent *t* comparisons mean the difference between biochemical profiles in *S. mansoni*-positive and *S. mansoni*-negative of study participants, Sanja town, northwest Ethiopia, 2019.

Parameter	*S. mansoni* positive (*N* = 110)	Control group (*N* = 110)	*P* value
	Mean ± SD	Mean ± SD	
Blood glucose (mg/dl)	99.71 ± 18.67	104.13 ± 16.64	0.065
Serum protein (g/dl)	68.7 ± 3.20	69.76 ± 3.0	0.012
AST (IU/L)	24.84 ± 2.19	23.86 ± 2.22	0.01
ALT (IU/L)	22.70 ± 4.66	21.15 ± 2.30	0.02
Cholesterol	149.08 ± 9.38	166.47 ± 18.44	<0.001

**Table 3:** Association of *S. mansoni* parasitic intensity with host hematological and biochemical profiles in Sanja town, northwest Ethiopia, 2019.

Parameter	Control group (0 epg)	Light (1–99 epg)	Moderate (100–399 epg)	Heavy (≥400)	*P* value
	Mean ± SD	Mean ± SD	Mean ± SD	Mean ± SD	
WBC (×10^3^/*μ*L)	7.81 ± 3.44	4.93 ± 1.48	4.04 ± 1.69	2.47 ± 1.15	<0.001
L (×10^3^/*μ*L)	2.48 ± 1.32	1.61 ± 0.61	1.28 ± .4971	0.93 ± 0.92	<0.001
Mixed (×10^3^/*μ*L)	1.55 ± 1.29	0.58 ± 0.50	0.51 ± 0.60	0.43 ± 0.40	<0.001
Granulocyte (×10^3^/*μ*L)	4.03 ± 2.07	2.85 ± 1.17	2.22 ± 1.39	1.10 ± 0.17	<0.001
RBC (×10^6^/*μ*L)	4.04 ± 0.65	4.21 ± 1.16	3.53 ± 0.39	2.52 ± 0.13	<0.001
Hgb (g/dl)	12.58 ± 1.67	12.04 ± 1.73	10.01 ± 1.19	8.30 ± 1.04	<0.001
PCV (%)	37.51 ± 5.11	35.94 ± 5.02	30.62 ± 3.46	24.87 ± 2.84	<0.001
MCV (*μ*m^3^)	86.45 ± 6.94	92.37 ± 10.71	93.25 ± 8.55	98.10 ± 5.89	<0.001
MCH (g/dL)	30.35 ± 2.95	29.65 ± 3.41	27.31 ± 2.96	26.20 ± 3.29	<0.001
MCHC (Pg)	34.80 ± 1.00	30.79 ± 4.06	28.68 ± 3.69	27.30 ± 5.72	<0.001
Blood glucose (mg/dl)	104.13 ± 16.63	107.15 ± 15.90	83.87 ± 13.16	75.00 ± 4.33	<0.001
Serum protein (g/dl)	69.76 ± 3.03	69.63 ± 2.84	66.69 ± 3.12	65.97 ± 1.15	<0.001
AST (U/L)	23.86 ± 2.22	24.28 ± 2.11	25.96 ± 1.82	27.43 ± 1.15	<0.001
ALT (U/L)	21.15 ± 2.27	22.30 ± 5.37	23.45 ± 2.32	24.93 ± 64	0.005
Total cholesterol (mg/dl)	166.47 ± 18.43	151.33 ± 9.06	144.15 ± 8.46	143.00 ± 5.00	<0.001

## Data Availability

All data are available within the article.
